# Typhoid Fever in Childhood

**Published:** 1888-10

**Authors:** F. Forchheimer

**Affiliations:** Cincinnati, Ohio


### TYPHOID FEVER IN CHILDHOOD.

BY F. FORCHHEIMER, M. D., CINCINNATI.

The doctor, who is Professor of Physiology, Medical College of Ohio, Physician to the Good Samaritan, Cincinnati and Children's hospitals, in a clinical lecture before the class at the Cincinnati Hospital, discussed this question, in brief, as follows:

*Gentlemen*—Please to note well in the history of this boy that we to-day bring before you, that he was taken ill on a certain day; that the day of the week and the month is given. In the majority of instances the disease begins suddenly in children, and this is one of the great characteristics with them. In the case of the adult the history is indefinite. The patient tells you that he has been feeling badly for a week or ten days. He cannot tell you the day on which he was taken sick. Cannot put his finger on it as the man with the pneumonia can with his pain, but spreads his whole hand out over a number of days as does the man who has a bronchitis. In children the disease comes on so suddenly and seriously that the doctor is usually called in the first 24 hours. The child was probably playing about in the morning, languid in the evening and quite ill by morning. It is the rarest thing in the world for this to occur in the adult.

The child does not locate the abdominal pain, but complains of bellyache, and when asked to tell where the pain is will pass his hand indefinitely over the whole surface of the abdomen. Careful examination to locate the pain will not find the abdomen at all tender. In children any pain in the abdominal organs will be referred to the epigastrium. Tenderness will be elicited on deep pressure in the iliac regions. Often we are only able to tell that there is pain by the child's crying or frowning. Children frequently suffer from insomnia in the beginning of the disease, which may alternate with drowsiness which is not very well marked. As a rule, the child does not sleep during the night, but is somnolent during the day.

*Symptoms as regards the nose.*—Epistaxis is common, but absent in many cases. Only 5 per cent. of my cases in this epidemic have had epistaxis. Not one of the 70 cases under my observation had it to any extent. This, as a rule, is the case; but in some epidemics it is quite severe and frequent. The nose is usually dry, and, as a rule, not dry enough to cause sneezing, which is generally absent. In this epidemic I have seen more than ever before, and Liebermeister's idea that if sneezing is present there is no typhoid fever does not always hold good.

The condition of the tongue I do not think is sufficient to risk a diagnosis upon. It is the same as in adults, large, coated with red borders and red lines in the center. We may find leptothrix and the remains of food.

Cough, bronchial catarrh, is present in nearly all cases.

*Abdominal symptoms.*—In children and infants, a great many suffer from constipation. I am fully aware that in this statement I differ from many authors. As a rule, diarrhœa occurs during the course of the disease, but it is usually not very severe. It is the pea-soup stool, with more or less pain, sometimes after the passage, but generally before. Constipation is, however, the rule, not diarrhœa.

Enlargement of the spleen occurs in a majority of cases, though it has not the importance in the diagnosis as with adults. There are well authenticated cases where the spleen was examined *post mortem* and found changed in structure, though not in size. Honoch reports a number where the spleen was normal; this has also been my experience.

Vomiting is present in most cases, very frequently in the inception, and we are often at a loss to know with what we are dealing for this reason.

The changes in the intestinal tract are by no means so severe as in the adult and are altogether different. The changes in Pyer's patches are not so extensive, more localized, intense, deep and as a rule do not extend into the large intestine. The child consequently suffers little from hemorrhage from the bowels. Only one out of my 70 cases had hemorrhage, and not one case of perforation occurred.

The great characteristic of typhoid fever in children is the deep and profound impression produced on the nervous system. We have nervousness, somnolence, wakefulness, headache and also changing of the disposition of the child. Those who were studious and agreeable before are now the opposite. After recovery they remain nervous, fidgety and shy, while sometimes the unstudious child becomes the reverse after the disease. This unbalancing of the nervous system in some cases lasts a number of years.

Circulatory system: As a rule the pulse bears no relation to the height of the temperature. With a temperature of 104.5 the pulse is not more rapid than with a temperature of 102. This is one of the secrets why the disease is not more frequently fatal in children; the heart is not so severely affected.

Complications are not so frequent in children. The per cent. was very small in this epidemic; the complication most often occurring in this epidemic was, that when the fever was breaking up, the child is suddenly attacked with aphasia. We have no explanation of the aphasia of typhoid fever; *post mortem* examination shows no change; it is probably there but not discovered. This aphasia lasts a week or ten days and the child begins to talk again. One sequel to typhoid fever, which occurs in children and not in adults, is tuberculosis. The case is protracted for six, eight or ten weeks, or it may be three months, and finally the child dies of tuberculosis of the intestines, acute miliary tuberculosis or tubercular meningitis. The only deaths from typhoid fever among children have been cases of this kind.

Prognosis is enormously favorable. In my 70 cases I have not lost one. The only exception to this general rule is among the newly born; the mortality during the first two months is very great. I do not believe entirely in this statement of the majority of authors; I think it incorrect. Up to the age of 12 years the mortality is hardly over 5 per cent. The death-rate in this hospital is between 6 and 7 per cent.

*Treatment.*—I believe in the possibility of aborting typhoid fever with calomel, and think I have done it six times in this epidemic, and have done it before. The whole trouble lies in the inability to prove this. If I get a case before the fifth day I always give a dose of calomel, and a large one, sometimes repeating it. I follow this up with a rather full dose of antipyrine, because it lessens pain and seems to have an antiseptic effect. I consider it of great importance to have two beds, one for the day and one for the night. It is necessary to have the largest, lightest and airiest room in the house. The diet should be absolutely fluid—no bread, no toast. I have seen hemorrhage occur from bread. The patient will complain bitterly of having nothing solid, nothing to chew. In these instances I find it very satisfactory to give tolu for them to chew. I frequently give a drop of the dilute nitro-muriatic acid everv hour. I give sustaining measures, mainly whisky, and in antipyretics avoid everything which will cause a collapse. Give the lukewarm bath.

